# Identification and antibiotic susceptibility of lactobacilli isolated from turkeys

**DOI:** 10.1186/s12866-018-1269-6

**Published:** 2018-10-29

**Authors:** Marta Dec, Anna Nowaczek, Dagmara Stępień-Pyśniak, Jacek Wawrzykowski, Renata Urban-Chmiel

**Affiliations:** 1 Department of Veterinary Prevention and Avian Diseases, Institute of Biological Bases of Animal Diseases, Faculty of Veterinary Medicine, University of Life Sciences in Lublin, Akademicka 12, 20-033 Lublin, Poland; 2 Department of Biochemistry, Faculty of Veterinary Medicine, University of Life Sciences in Lublin, Akademicka 12, 20-033 Lublin, Poland

**Keywords:** antibiotic susceptibility, *Lactobacillus*, poultry, resistance genes

## Abstract

**Background:**

The aim of this study was to identify *Lactobacillus* isolates derived from turkeys from six Polish farms and to characterize their phenotypic and genotypic antibiotic resistance profiles.

**Results:**

Among 62 isolates identified by MALDI-TOF mass spectrometry and restriction analysis of 16S rDNA, the dominant species was *L. salivarius* (35%), followed by *L. crispatus* (21%), *L. ingluviei* (14.5%) and *L. johnsonii* (10%). A high prevalence of resistance to tetracycline (68% resistant isolates), lincomycin (64.5%) and enrofloxacin (60%) among the lactobacilli tested was observed. Fewer than 50% isolates were resistant to ampicillin (47%), erythromycin (45%), streptomycin (31%), chloramphenicol (29%) and gentamicin (10%). As many as 64,5% of the isolates showed multidrug resistance. High MIC values for ampicillin (≥64 μg/ml) were usually accompanied by elevated MICs for cephalosporins (≥16 μg/ml) and high MICs for tiamulin, i.e. ≥32 μg/ml, were noted in most of the turkey lactobacilli (61%). The occurrence of resistance genes was associated with phenotypic resistance, with the exception of five phenotypically susceptible isolates that contained the *tetM*, *tetL*, *ermC*, *ermB* or *cat* genes. The most frequently identified were *ermB* (45% isolates), *tetL* (40%), *tetW* (37%) and *tetM* (29%), and the occurrence of *lnuA* (18%), *cat* (10%), *ermC* (6%), *ant(6)-Ia* (5%) and *aadE* (5%) was less frequent. The mechanism of ampicillin resistance has not been elucidated, but the results of nitrocefin test confirmed that it is not involved in the production of beta-lactamases.

**Conclusions:**

The high rate of antibiotic resistance observed in this study indicates the need to implement the principles of rational use of antibiotics in poultry. The presence of transmissible resistant genes in lactobacilli may contribute to the development of antibiotic resistant pathogenic strains that pose a threat to both poultry and consumers. The results of these studies may be useful for committees providing guidance on antibiotic susceptibility of microorganisms in order to revise and supplement current microbiological cut-offs values within the genus *Lactobacillus*.

**Electronic supplementary material:**

The online version of this article (10.1186/s12866-018-1269-6) contains supplementary material, which is available to authorized users.

## Background

Bacteria of the genus *Lactobacillus* are Gram-positive, aerotolerant or anaerobic catalase-negative rods or coccobacilli with a G+C content usually below 54 mol% [1]. They are the most numerous group of lactic acid bacteria (LAB), with 228 species described to date (July 2018) [2]. Based on the 16S rRNA gene sequence, lactobacilli have been divided into 15 large phylogenetic groups, 7 small groups of two species each, and 7 groups represented by single *Lactobacillus* species [3]. Due to their high nutritional requirements, lactobacilli colonize environments rich in carbohydrate-containing substances: they are found on plants or material of plant origin, in fermented food products, or in association with mucous membranes of humans and animals. *Lactobacillus* species found in the gastrointestinal tract (GIT) have received a great deal of attention due to their health-promoting properties. By acidifying the intestines and through other antimicrobial mechanisms, they help to eliminate unfavourable microflora and maintain a natural microbial balance. Their positive effect on host also includes improved digestion and adsorption of nutrients, modulation of immune response and reduction of toxic and mutagenic compounds in the gut and serum cholesterol level [4]. Selected strains of *Lactobacillus* are used as probiotics for humans and animals, and interest in applications for these bacteria continues to grow.

The poultry industry is one of the fastest-growing segments of the livestock sector worldwide. Poland currently remains the largest producer and leading exporter of poultry meat in the European Union. Domestic production consists mainly of broiler chickens, but in recent years there has also been an increase in turkey meat production, which is currently estimated at over 400,000 tonnes per year [5]. Broiler turkey meat is valued all over the world for its delicate taste, low fat content and high levels of valuable protein. However, turkeys are considered difficult to raise, especially in the early stages, due to their sensitivity to adverse environmental conditions and high nutritional requirements [6]. They are also susceptible to many diseases, including bacterial infections, which can be primary or secondary [7].

These problems contribute to the frequent use of antibiotic drugs in turkey farming. Unfortunately, in many cases antibiotic therapy is not used rationally, which greatly contributes to the development of antibiotic resistance among bacteria, both pathogenic and commensal. The GIT, which is inhabited by a large number of diverse bacteria, is considered a reservoir of resistance genes [8]. In such a microbiologically rich environment, it may be possible to exchange genetic material between pathogenic, potentially pathogenic and non-pathogenic bacteria. It has been demonstrated that genetic resistance determinants located on mobile elements, such as plasmids, can be transferred horizontally not only between different *Lactobacillus* species but also to other species such as potentially pathogenic enterococci [9].

Bacteria, including lactobacilli, become resistant not only via the acquisition of resistance genes from other organisms through horizontal transfer (conjugation, transformation and transduction), but also by *de novo* mutation. The most well-known mechanisms of bacterial resistance include: i) modifications of the antimicrobial target (decreasing the affinity for the drug), ii) decreased permeability of a bacterial cell wall for drug, iii) activation of efflux mechanisms that extrude the medicine out of the cell, iii) production enzymes that destroy drug’s structure, iv) development of an alternative metabolic pathway to those inhibited by the drug [10].

More and newer resistance mechanisms are emerging and spreading globally. Antibiotic-resistant strains propagated in these livestock pose a threat to animals and could be widely disseminated via the food chain. Commensal microflora of the GIT of poultry are frequently present in fresh poultry meat products and may serve as reservoirs of resistant genes that could be transferred to bacterial pathogens of people. Drug-resistant strains cause infections difficult to control and therefore the development of resistance in bacteria is associated with elevated morbidity and mortality rates and increased treatment costs in both animals and humans [11].

Lactobacilli are currently widely used as probiotic supplements, and the strains selected have to meet several criteria, including antibiotic susceptibility. In line with EFSA's FEEDAP Panel (European Food Safety Authority Panel on Additives and Products or Substances used in Animal Feed) recommendations, to differentiate resistant strains from susceptible ones, MIC values should be determined for the number of antibiotics and chemiotherapeutics. Strains showing acquired resistance should not be used as feed additives except when the basis or resistance is chromosomal mutation [12].

The aim of this study was to identify *Lactobacillus* isolates derived from turkeys, determine their antibiotic susceptibility and detect drug-resistance genes. To the best of our knowledge, no studies have previously been undertaken on characterization of turkey lactobacilli.

## Methods

### Isolation of lactobacilli

*Lactobacillus* bacteria were isolated from the fresh faeces or cloacae of 22 healthy turkeys from 6 large-scale poultry farms located in different parts of Poland (Lubelskie, Warmińsko-Mazurskie and Wielkopolskie voivodships). Samples were collected during the period from 2012 to 2013, numbering three or four per farm. The age of the birds ranged from 1 week to 15 weeks. No probiotics were administered on any farm.

Bacteria were isolated on MRS (Man, Rogosa and Sharp) medium (BTL, Poland) at 37°C for 48 h in 5% CO_2_. All isolates were Gram-positive and catalase-negative. The strains were kept in deMan Rogosa Sharpe broth (MRS, BTL, Poland) containing ~20% glycerol at −80°C.

### Species identification using MALDI-TOF MS

The MALDI-TOF-MS analysis was done using a UltrafleXtreme MALDI-TOF mass spectrometer (Bruker, Germany). Bacterial colonies were smeared onto stainless steel MALDI MS target plate and overlaid with 1 μL of 70% formic acid before adding 1 μL of matrix solution. The analysis of the microbial mass spectra was carried out using MALDI Biotyper 3.0 software (Bruker, Germany) [13].

The results of pattern matching were expressed as numerical score ranging from 0 to 3.00 according to the criteria recommended by Bruker: log(score) ≥2.30 (2.30–3.00) - secured isolate identification at species level, log(score) 2.00 to 2.29 - probable identification at the species level, log(score 1.70 to 1.99 indicates identification at genus level and score >1.70 no reliable identification (http://www.bruker.com). The triplicate spot scores were recorded and the highest log(scores) (1.700-3.000) were considered reliable. If the difference between the log values (score) of the two best runs was less than 0.30, the identification was considered non-significant [13].

### Identification of lactobacilli by 16S-ARDRA

Nine isolates for which definitive species identification was not obtained using MALDI-TOF MS (*L. johnsonii*/*L. gasseri*, *L. crispatus/L. ultunensis*, or *L. oris/L. antri*) were identified using Amplified Ribosomal DNA Restriction Analysis of 16S rDNA (16S-ARDRA). Isolation of bacterial genomic DNA from lactobacilli and amplification of 16S rDNA were performed according to the protocol described in our previous work [13].

Eight reference *Lactobacillus* strains were used in the experiment: *L. antri* LMG 22111, *L. crispatus* LMG 9479, *L. gasseri* LMG 13134, *L. gasseri* ATCC 19992, *L. johnsonii* LMG 18195, *L. johnsonii* LMG 9436, *L. oris* LMG 9848 and *L. ultunensis* LMG 22117. The 16S rDNA amplicons were digested with 3 restriction enzymes – *Alu*I, *Mse*I and *Mbo*I (Thermo Scientific, USA), which were selected on the basis of *in silico* analysis using CLC Main Workbench software (Qiagen) and the 16S rDNA nucleotide sequences of the *Lactobacillus* strains, deposited in GenBank.

Ten μl of PCR product was digested in 12.7 μl of restriction enzyme buffer containing 0.7 μl of enzyme (initial concentration of each restriction enzyme 10 U/μl) and left to react at 65°C (for *Mse*I) or at 37°C (for *Alu*I and *Mbo*I) for 4 h. DNA electrophoresis and analysis of restriction profiles were carried out as described in a previous work [13].

### Determination of minimal inhibitory concentration

Antibiotic susceptibility of all bacterial isolates was determined by the broth microdilution procedure [14], using the LAB susceptibility test medium (LSM) (Iso Sensitest broth containing 10% of MRS) recommended by ISO 10932/IDF 223 [15]. All dry powder antibiotics were purchased in Sigma-Aldrich (Poland), with the exception of ampicillin, which was obtained from Roth (USA). As the source of enrofloxacin and tiamulin, ready-made solutions of drugs were used (Enrocin, 50 mg/ml, Vet-Agro, Poland and Biomutin, 200 mg/ml, BIOWET DRWALEW S.A. Poland). Cephalosporins were dissolved in water and stock solutions for other antimicrobial agents were prepared as described in our previous work [16].

Fresh cultures grown overnight on LSM medium were used to prepare the bacterial suspensions in 0.9% NaCl (final optical density at 600 nm was 0.5). Then, 50 μl of a bacterial suspension previously diluted 1:500 in an LSM medium with 50 μl of the antibiotic solution were mixed together on a microplate. The plates were incubated 48 h at 37°C in 5% CO_2_ and then the MICs were read visually as the lowest concentration of antimicrobial substance that inhibited the growth of bacteria. *Enterococcus faecalis* ATCC 29212, *Lactobacillus johnsonii* ATCC 33200 and *S. aureus* ATCC 29213 were included as a quality control strains (to control of antibiotics potency and quality of medium) [15, 17, 18]. *Lactobacillus johnsonii* ATCC 33200 were run in parallel with wild-type *Lactobacillus* isolates in each trial.

The EFSA's FEEDAP Panel guidelines [12] were used to interpret the results ampicillin, tetracycline, erythromycin, streptomycin, gentamicin and chloramphenicol. For lincomycin and enrofloxacin, the criteria suggested earlier by Cauwers et al [19] and Dec et al. [16] were adapted. The bacteria were considered resistant if the MIC was ≥64 μg/ml for lincomycin and enrofloxacin. No cut-off values for tiamulin, cephalothin, cefuroxime and ceftiofur were proposed due to insufficient number of isolates and no bimodal MIC distribution for most *Lactobacillus* species.

### Detection of resistance genes

To detect resistance genes and *Tn916/Tn1545*-like transposon (integrase gene *Int-Tn*), 23 gene-specific PCR primer pairs were used (Table 1). The PCR mixture for detection of single resistance genes was prepared in a 25 μl volume containing 2.5 μl 10x Dream Taq Buffer, 0.12 μl Dream Taq DNA polymerase (5 U/ml, Thermo Fisher Scientific), 1.25 μl 8 mM dNTPs mix (Blirt, Poland), 0.8 μl of each of two primers (10 pmol/μl, Sigma-Aldrich, Poland), 1 μl template DNA (~20 ng) and 18.5 μl water (Sigma-Aldrich, Poland). Multiplex PCR for detection of some tetracycline, macrolide and aminoglycoside resistance genes (Table 1) was carried out following previously described protocols [20, 21].

**Table 1:** Primers used for detection of selected antibiotic resistance genes

Determining resistance to	Target gene	Primer sequence (5’→3’)	Amplicon size (bp)	Annealing temperature (°C)	Reference
tetracyclines	*tetM*	GTG GAC AAA GGT ACA ACG AG CGG TAA AGT TCG TCA CAC AC	406	60	[20]
	*tetK*	GAT CAA TTG TAG CTT TAG GTG AAG G TTT TGT TGA TTT ACC AGG TAC CAT T	155	60	
	*tetL*	TGG TGG AAT GAT AGC CCA TT CAG GAA TGA CAG CAC GCT AA	229	60	
	*tetO*	AAC TTA GGC ATT CTG GCT CAC TCC CAC TGT TCC ATA TCG TCA	515	60	
	*tetW*	GAG AGC CTG CTA TAT GCC AGC GGG CGT ATC CAC AAT GTT AAC	168	64	[50]
macrolides and lincosamides	*ermA*	CCC GAA AAA TAC GCA AAA TTT CAT CCC TGT TTA CCC ATT TAT AAA CG	590	60	[20]
	*ermB*	TGG TAT TCC AAA TGC GTA ATG CTG TGG TAT GGC GGG TAA GT	745	60	
	*mefA/E*	CAA TAT GGG CAG GGC AAG AAG CTG TTC CAA TGC TAC GC	317	60	
	*ermC*	AAT CGT CAA TTC CTG CAT GT TAATCGTGGAATACGGGTTTG	299	58	[35]
	*lnuA*	GGT GGC TGG GGG GTA GAT GTA TTA ACT GG GCT TCT TTT GAA ATA CAT GGT ATT TTT CGA TC	323	61	[51]
aminoglycosides	*aac(6’)-Ie-aph(2”)-Ia*	CAG AGC CTT GGG AAG ATG AAG CCT CGT GTA ATT CAT GTT CTG GC	348	56	[21]
	*aph3IIIa*	GGC TAA AAT GAG AAT ATC ACC GG CTT TAA AAA ATC ATA CAG CTC GCG	523		
	*ant(4’)-Ia*	CAA ACT GCT AAA TCG GTA GAA GCC GGA AAG TTG ACC AGA CAT TAC GAA CT	294		
	*aph(2”)-Ic*	CCA CAA TGA TAA TGA CTC AGT TCC C CCA CAG CTT CCG ATA GCA AGA G	444		
	*aph(2”)-Id*	GTG GTT TTT ACA GGA ATG CCA TC CCC TCT TCA TAC CAA TCC ATA TAA CC	641		
	*ant(6)-Ia*	CGG GAG AAT GGG AGA CTT TG CTG TGG CTC CAC AAT CTG AT	563	56	[52]
	*aac(6’)-Ii*	TGGCCGGAAGAATATGGAGA GCATTTGGTAAGACACCTACG	410	55	
	*aadE*	ATG GAA TTA TTC CCA CCT GA TCA AAA CCC CTA TTA AAG CC	1060	51	[43]
chloramphenicol	*cat*	TAA GGT TAT TGG GAT AAG TTA GCA TGR TAA CCA TCA CAW AC	340	54	[23]
tiamulin	*lsaE*	TGT CAA ATG GTG AGC AAA CG TGT AAA ACG GCT TCC TGA TG	496	54	[53]
penicillins	*blaZ*	ACT TCA ACA CCT GCT GCT TTC TAG GTT CAG ATT GGC CCT TAG	240	60	[54]
	*mecA*	AGT TCT GCA GTA CCG GAT TTG C AAA ATC GAT GGT AAA GGT TGG C	533	55	[55]
	*int-Tn* (Tn*916/*Tn*1545)*	GCGTGATTGTATCTCACT GACGCTCCTGTTGCTTCT	1028	55	[49]

DNA amplification was performed using an Eppendorf Mastercycler at following conditions: 5 min at 95°C, 30 cycles with 40 s at 95°C, 40 s at 50-64°C (according to the annealing temperature for the individual primers; Table 1) and 75 s at 72°C and 8 min of final extension at 72°C. PCR products (8 μl) were analysed by electrophoresis (100 V) on 2% agarose gels containing ethidium bromide (0.5 ug/ml).

As a positive control there were used *Lactobacillus* and *Enterococcus* wild isolates containing resistance genes as well as reference strain *Stapylococcus aureus* ATCC 33591 (Table 2). The PCR products obtained for representative wild-type strains were sequenced, and the results of comparative analysis using the NCBI BLAST algorithm (http://www.ncbi.nlm.nih.gov/Blast.cgi) confirmed that the amplicons are counterparts of the resistance genes (Additional file 1).

**Table 2:** Lactic acid bacteria used as positive controls during an experiment to detect resistance genes

Isolate	Source	Genotype	Reference
*L. salivarius* 3a	chicken	*lsaE, aac(6’)-Ie-aph(2”)-Ia, aadE, ant(6)-Ia*	GeneBank Ac. No. KY924692
*S. aureus* ATCC 33591	clinical isolate	*blaZ, mecA*	GeneBank Ac. No. KY264166.1, FJ809758.1
*L. salivarius* 3aI	turkey	*tetL, tetM, ermB, ermC*	The sequences of amplicons reflecting the resistance genes and the results of the comparative analysis with the reference sequences deposited at GenBank were showed in the Additional file 1.
*L. salivarius* 5aI	turkey	*ant(6)-Ia, aadE, ermC*	
*L. salivarius* 27eCh	chicken	*aph(2”)-Ic, tetW, ermB, ant(6)-Ia*	
*L. salivarius* 30aI	turkey	*tetL, tetM, ermB, int-Tn*	
*L. ingluviei* 22eI	turkey	*tetL, tetW, lnuA, ermB, cat*	
*E. faecalis* 3W	wolf	*tetM, ermB*, *msrA/B*, *aph3IIIa, ant(4’)-Ia, aac(6’)-Ie-aph(2”)-Ia, int-Tn*	
*E. faecium* 24W	wolf	*aac(6’)-Ii, tetM, msrA/B,*	
*E. faecium* 60	woodpecker	*aph(2”)-Id, ant(6)-Ia, aph(3’)-IIIa, tetM, ermB, msrA/B*	
*E. faecalis* 140	chicken	*tetO, ant(4')-Ia, int-Tn*	

### Nitrocefin test

In this test *Lactobacillus* isolates displaying phenotypic resistance to ampicillin were used. A loopful of overnight culture grown on MRS agar around the ampicillin disks (induction of β-lactamase production) was smeared on the moisturized nitrocefin strips (DIAGNOSTICS Inc., Slovak Republic). If red color appeared on the strips in 15 min, bacteria were considered as beta-lactamase positive. Three isolates of *E. coli* in which the *bla*_TEM-1_ gene was previously detected [22] were used as positive control.

## Results

### Identification of lactobacilli

A total of 62 isolates with rod-shaped morphology were classified as bacteria of the genus *Lactobacillus* with a Biotyper log(score) equal to or greater than 1.70. For 4 (6%) isolates the log(score) was 2.3-3.0, for 34 (55%) it was 2.00-2.29, and for 24 (39%) it was 1.70-1.99 (Additional file 2).

For 53 isolates (85%) the best matches (1.700-3.000) were considered to be correct species identification. Identification of the remaining 9 strains was considered ambiguous because the first and second best matches (log(score) 1.7-2.3) indicated different species, and the difference between their log(score) values were less than 0.30. For 5 of these samples the best match indicated *L. johnsonii* and the second best match *L. gasseri*, for 2 samples the best match indicated *L. crispatus* and the second best match *L. ultunensis*, and for another 2 samples the best match indicated *L. oris* and the second best match *L. antri.*

Among the 62 isolates identified to the species level (log(score) 1.7-3.0), the species identified were *L. salivarius* – 22 strains, *L. crispatus* – 11, *L. crispatus*/*L. ultunensis* – 2, *L. ingluviei* – 9, *L. johnsonii -*1, *L. johnsonii/L. gasseri* – 5, *L. oris* – 3, *L. oris/L. antri –* 2, *L. saerimneri –* 3, *L. agilis* – 2, and *L. reuteri* – 2 strains (Additional file 2).

### Identification of lactobacilli using 16S-ARDRA

Analysis of the electrophoretic profiles obtained by digestion of 16S rDNA amplicons with selected restriction enzymes showed that the use of *Mse*I allowed for differentiation between *L. gasseri* and *L. johnsonii* but not between *L. crispatus* and *L. ultunensis* or between *L. oris* and *L.antri*. Different electrophoretic profiles for *L. crispatus* and *L. ultunensis* were obtained only following digestion with *Mbo*I, and differences between *L. oris* and *L. antri* appeared after digestion with *Alu*I.

**Fig. 1:**
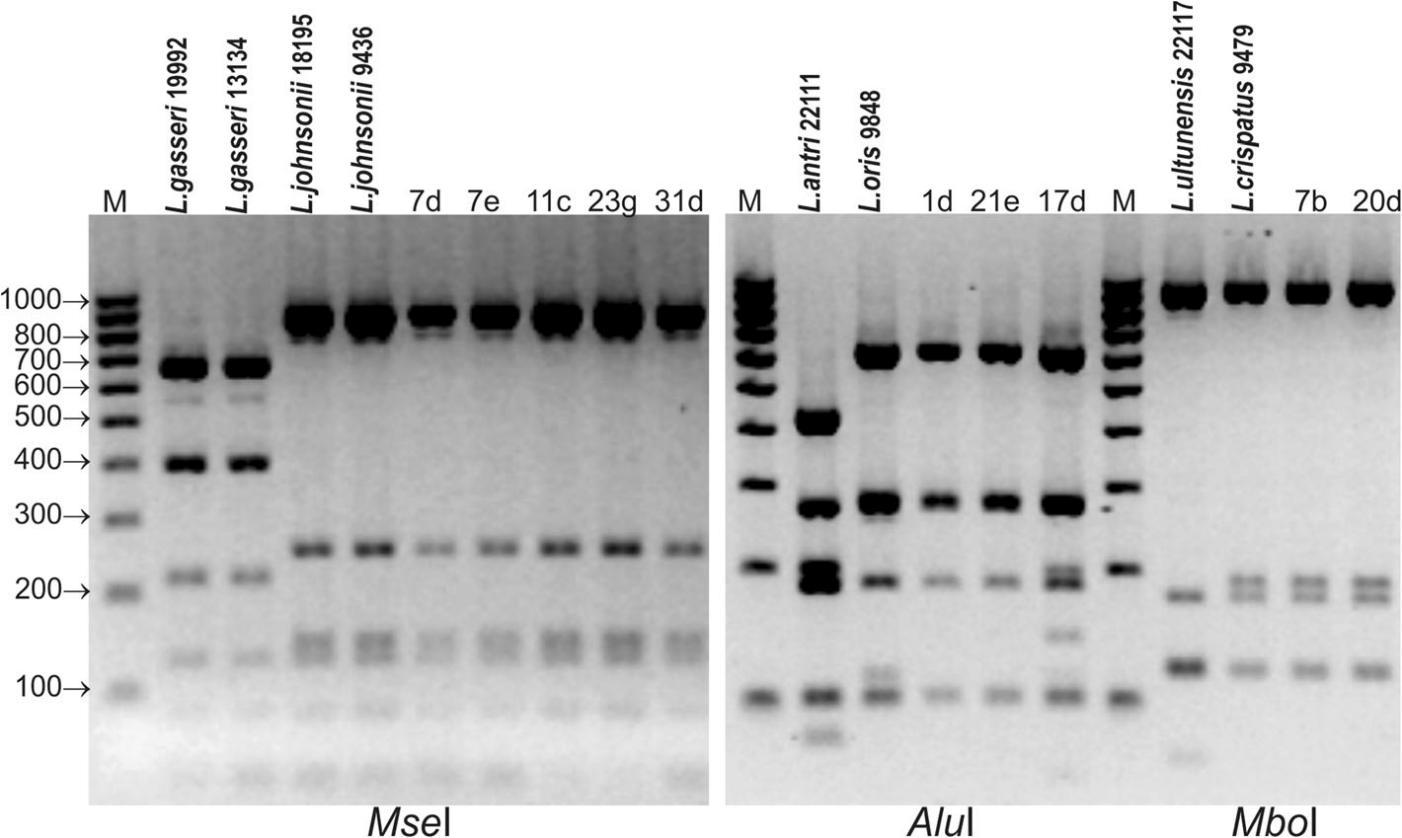
ARDRA patterns of reference and wild poultry *Lactobacillus* strains obtained by digestion of 16S rDNA amplicons with *Mse*I, *Mbo*I and *Alu*I

Analysis of the electrophoretic restriction profiles showed that all the strains previously identified in MALDI-TOF MS as *L. johnsonii*/*L. gasseri* belonged to the species *L. johnsonii*, 2 isolates identified as *L. crispatus/L. ultunensis* belonged to the species *L. crispatus*, and 3 species determined as *L. oris/L. antri* belonged to the species *L. oris* (Fig. 1).

The electrophoretic profiles of digested 16S rDNA amplicons contained 3-6 restriction fragments ranging from 82 to 920 bp (Additional file 3).

### Antimicrobial susceptibility testing

The MIC of 12 antibiotic agents was analysed for 62 *Lactobacillus* isolates from turkeys. The MIC range was 0.25->64 μg/ml for ampicillin, 0.25-64 μg/ml for cephalothin, ≤0.125->64 μg/ml for cefuroxime, ≤0.125-64 μg/ml for ceftiofur, 2-512 μg/ml for tetracycline, ≤0.25->64 μg/ml for erythromycin, ≤1->1,024 μg/ml for lincomycin, ≤1->1,024 μg/ml for streptomycin, 2->128 μg/ml for gentamycin, 1-128 μg/ml for chloramphenicol, ≤0.5->256 μg/ml for tiamulin, and ≤1-256 μg/ml for enrofloxacin (Table 3). According to the established criteria, 68% of isolates were resistant to tetracycline, 64.5% to lincomycin, 60% to enrofloxacin, 47% to ampicillin, 45% to erythromycin, 31% to streptomycin, 29% to chloramphenicol, and 10% to gentamicin (Table 4). High MIC values for ampicillin (≥64 μg/ml) recorded for 15 (24%) isolates (*L. salivarius* and *L. crispatus*) were usually accompanied by elevated MICs for cephalosporins (≥16 μg/ml) (Additional file 2). As much as 90% *Lactobacillus* isolates showed a MICs range for tiamulin from 2 to 256 μg/μl, and for 10% (6) isolates (1 *L. reuteri*, 2 *L. johnsonii* and 3 *L. ingluviei*) we recorded a particularly low tiamulin MICs, ie. ≤0.5 μg/ml (Table 3). Multiple-drug resistance (resistance to at least 3 groups of antimicrobial agents) was observed for 64.5% of lactobacilli, and 43.5% isolates showed cross-resistance between erythromycin and lincomycin. Simultaneous resistance to streptomycin and gentamicin was recorded for 6% of isolates (Table 4). Only three *L. ingluviei* isolates (I22b, I23c and I24b), derived from the same farm, showed susceptibility to all the drugs tested, and their MIC values for tiamulin were as low as ≤0.5 μg/ml (Table 3).

**Table 3:**
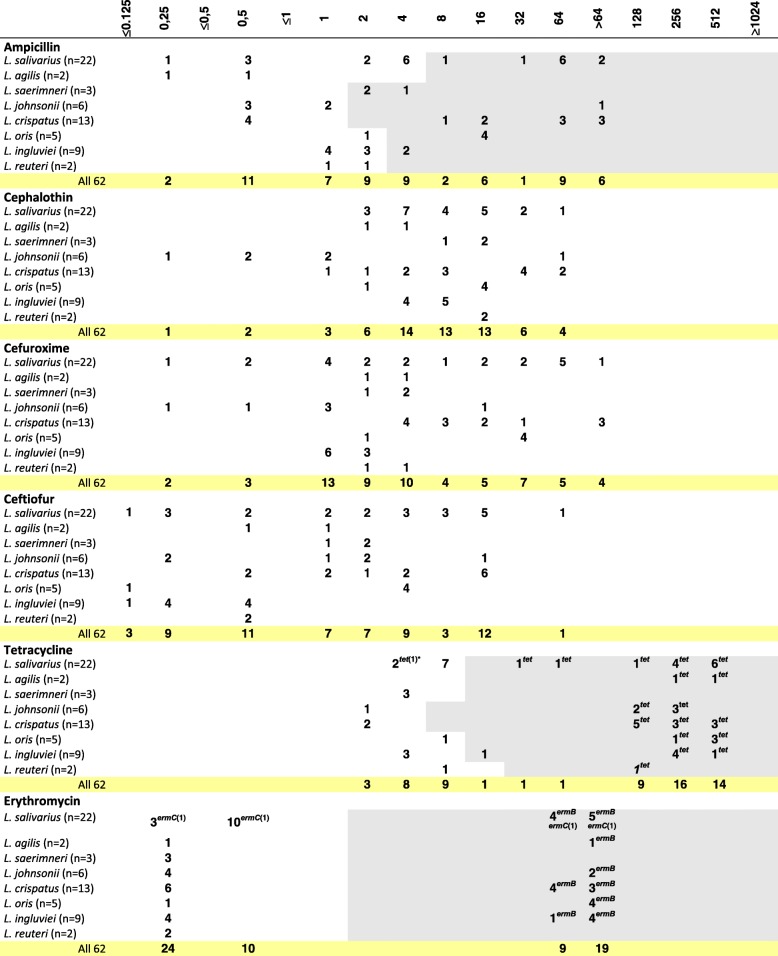

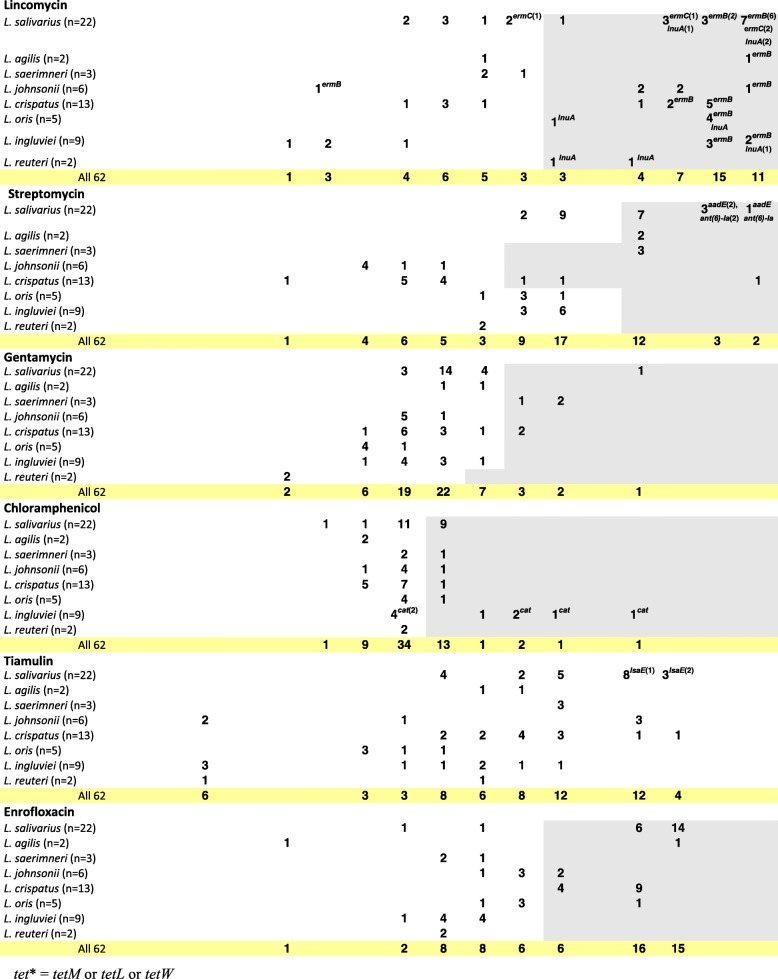
Distribution of MICs of antibiotics among various Lactobacillus species of turkey origin

Fragments highlighted in grey indicate MIC cut-off values (μg/mL) as indicated in the Methods. The number of isolates carrying the gene in question is given in brackets after the name of the gene. The absence of a number following the name of the gene means that all isolates contain the gene

tet* = *tetM* or *tetL* or *tetW*

**Table 4:** Number of resistant *Lactobacillus* strains determined on the basis of MIC breakpoints established for the antibiotics

	Number of strains												
	do not showing any resistance	resistant to one drug only	with multidrug resistance^a^	displayed resistance against									
				ampicillin (MIC≥2 or 4 or 8 μg/ml)	tetracycline (MIC≥8 or 16 or 32 μg/ml)	erythromycin (MIC≥2 μg/ml)	lincomycin (MIC≥64 μg/ml)	streptomycin (MIC≥32 or 128 μg/ml)	gentamycin (MIC≥32 μg/ml)	chloramphenicol (MIC≥8 μg/ml)	enrofloxacin (MIC≥64 μg/ml)	erythromycin and lincomycin	streptomycin and gentamycin
*L. salivarius* (*n*=22)	0	2 (9%)	15 (68%)	10 (45%)	13 (59%)	9 (41%)	14 (64%)	11 (50%)	1 (4.5%)	9 (41%)	20 (91%)	9 (41%)	1 (4.5%)
*L. agilis* (*n*=2)	0	0	2 (100%)	0	2 (100%)	1 (50%)	1 (50%)	2 (100%)	0	0	1 (50%)	1 (50%)	0
*L. saerimneri* (*n*=3)	0	0	1 (67%)	3 (100%)	0	0	0	3 (100%)	3 (100%)	1 (33%)	0	0	3 (100%)
*L. johnsoni* (*n*=6)	0	1 (17%)	3 (50%)	1 (17%)	5 (83%)	2 (33%)	5 (83%)	0	0	1 (17%)	2 (33%)	1 (17%)	0
*L. crispatus* (*n*=13)	0	0	10 (77%)	9 (69%)	11 (85%)	7 (54%)	8 (61.5%)	3 (23%)	2 (15%)	1 (8%)	13 (100%)	7 (38%)	0
*L. oris* (*n*=5)	0	1 (20%)	4 (80%)	4 (80%)	4 (80%)	4 (80%)	5 (100%)	0	0	1 (20%)	1 (20%)	4 (80%)	0
*L. ingluviei* (*n*=9)	3 (33%)	0	5 (55.5%)	2 (22%)	6 (67%)	5 (55.5%)	5 (55.5%)	0	0	5 (55.5%)	0	5 (55.5%)	0
*L. reuteri* (*n*=2)	0	1 (50%)	0	0	1 (50%)	0	2 (100%)	0	0	0	0	0	0
Total: 62	**3** **(5%)**	**5** **(8%)**	**40** **(64.5%)**	**29** **(47%)**	**42** **(68%)**	**28** **(45%)**	**40** **(64.5%)**	**19** **(31%)**	**6** **(10%)**	**18** **(29%)**	**37** **(60%)**	**27** **(43.5%)**	**4** **(6%)**

^a^resistant to at least 3 groups of antimicrobial agents (the analysis excluded tiamulin, cephalothin, cefuroxime and ceftiofur for which the breakpoints has not been established)

Clear bimodal distribution of MICs indicative of acquired resistance was observed for erythromycin, lincomycin and tetracycline (for all species beside *L. salivarius*). Bimodal distribution was also noted for ampicillin MICs for *L. johnsonii* and *L. crispatus* isolates and MICs of enrofloxacin for *L. salivarius*, *L. agilis* and *L. oris*. Regarding the susceptibility of *L. salivarius* to ampicillin and tetracycline, we noted three MIC ranges, which could indicate the presence of sensitive, intermediate and resistant strains. In the case of aminoglycosides, chloramphenicol and tiamulin, distribution of MIC values was unimodal for most *Lactobacillus* species (Table 3).

### Detection of antibiotic resistance genes

Of the 23 considered resistance genes, 10 were detected in the tested lactobacilli. We found *tet* genes conferring resistance to tetracyclines in 42 (68%) isolates, including 41 phenotypically resistant to tetracycline and one susceptible strain (it contained *tetM* and *tetL* genes). The most frequently identified *tet* gene was *tet*L, which was observed in 40% of isolates, followed by *tetW* (37%) and *tetM* (29%) (Table 5). The presence of individual *tet* genes seems to be correlated with the species. The *tetM* gene was found in *L. salivarius*, *L. crispatus* and *L. agilis*; *tetL* was detected in *L. salivarius*, *L. agilis*, *L. crispatus*, *L. oris*, and in one isolate of *L. ingluviei*. The presence of the *tet*W gene was unique for the species belonging to the phylogenetic group of *L. delbrueckii* (*L. johnsonii* and *L. crispatus*) and *L. reuteri* (*L. oris*, *L. ingluviei* and *L. reuteri*). The co-occurrence of *tetM* and *tetL* was characteristic for *L. salivarius*, *L. agilis* and *L. crispatus*. The *tetL* and *tetW* genes were present simultaneously only in *L. crispatus*, *L. oris* and *L. ingluviei* isolates.

**Table 5:** Number of *Lactobacillus* strains carrying resistance genes^a^

Resistant gene →	*tetL*	*tetM*	*tetW*	*ermB*	*ermC*	*lnuA*	*tetL+ tetM*	*tetL+ tetM + ermB*	*tetW + ermB*	*ermB + lnuA*	*cat*	*ant(6)-Ia (aadE)*	*lsaE*	*lsaE + ant(6)-Ia* (*aadE*)	*int-Tn* (Tn*916/*Tn*1545)*
*L. salivarius* (*n*=22)	14 (64%)	12 (54.5%)	0	9 (41%)	4 (18%)	3 (14%)	12 (54.5%)	8 (36%)	0	2 (9%)	0	3 (14%)	3 (14%)	3 (14%)	3 (14%)
*L. agilis* (*n*=2)	2 (100%)	2 (100%)	0	1 (50%)	0	0	2 (100%)	1 (50%)	0	0	0	0	0	0	0
*L. saerimneri* (*n*=3)	0	0	0	0	0	0	0	0	0	0	0	0	0	0	0
*L. johnsonii* (*n*=6)	0	0	5 (83%)	2 (33%)	0	0	0	0	2 (33%)	0	0	0	0	0	0
*L. crispatus* (*n*=13)	4 (31%)	4 (31%)	8 (61.5%)	7 (54%)	0	0	4 (31%)	2 (15%)	4 (31%)	0	0	0	0	0	0
*L. oris* (*n*=5)	4 (80%)	0	4 (80%)	4 (80%)	0	5 (100%)	0	0	4 (80%)	4 (80%)	0	0	0	0	0
*L. ingluviei* (*n*=9)	1 (11%)	0	5 (55.5%)	5 (55.5%)	0	1 (11%)	0	0	5 (55.5%)	1 (11%)	6 (67%)	0	0	0	0
*L. reuteri* (*n*=2)	0	0	1 (50%)	0	0	2 (100%)	0	0	0	0	0	0	0	0	0
Total: 62	**25** **(40%)**	**18** **(29%)**	**23** **(37%)**	**28** **(45%)**	**4** **(7%)**	**11** **(18%)**	**18** **(29%)**	**11** **(18%)**	**15** **(24%)**	**7** **(11%)**	**6** **(10%)**	**3** **(5%)**	**3** **(5%)**	**3** **(5%)**	**3** **(5%)**

^a^- none of the isolate contained the resistance genes: *tetK, tetO, ermA, mefA/E, msrC, blaZ, mecA, aph(3’)-IIIa, aac(6’)-Ie-aph(2”)-Ia, aph(2”)-Ic, aph(2”)-Id, ant(4’)-Ia and aac(6’)-Ii*

Among the genes coding for resistance to macrolides and lincosamides, the most frequent was *ermB* (in 45% of isolates), rarely *ermC* (6%) and *lnuA* (18%). The rRNA methylase *ermB* gene was detected in all isolates resistant to erythromycin (27 isolates with MIC≥64 μg/ml) and in one isolate with a susceptible phenotype (*L. salivarius* 21b, MIC=0.5 μg/ml) (Tables 3, 5). The *ermC* gene (encoding methylase) was detected in 4 isolates of *L. salivarius*, including 2 phenotypically resistant to erythromycin and 2 susceptible to this antibiotic. Three of these *ermC*-positive isolates were resistant to lincomycin. The *lnuA* gene (encoding lincosamide *O*-nucleotidyltransferase) was detected in 11 strains, all of which were resistant to lincomycin (Table 3).

Among genes determining resistance to aminoglycoside antibiotics, *ant(6)-Ia* and *aadE* encoding ANT(6) adenyltransferases were detected. They occurred simultaneously in 3 isolates (5%) of *L. salivarius* showing resistance to streptomycin (MIC≤512 μg/ml). The results of the sequence analysis of PCR products indicated that *aadE* and *ant(6)-Ia* are the same gene detected by different primers (Additional file 1).

The *cat* gene encoding chloramphenicol acetyltransferase, which converts chloramphenicol to inactive diacetyl chloramphenicol [23], was present in 6 isolates (11%) of *L. ingluviei*, 4 of which were resistant to chloramphenicol (MIC≤8 μg/ml) (Tables 3, 5).

The *lsaE* gene coding for multidrug efflux pumps was present in 3 isolates of *L. salivarius* with high MIC values for tiamulin, i.e. >256 μg/ml (Tables 3, 5). These *lsaE*-positive isolates also contained the *aadE* or *ant(6)-Ia* gene conferring resistance to streptomycin.

None of the *Lactobacillus* isolates contained the *tetK*, *tetO*, *ermA*, *mefA/E, blaZ*, *mecA*, *aph(3’)-IIIa*, *aac(6’)-Ie-aph(2”)-Ia, aph(2”)-Ic, aph(2”)-Id*, *ant(4’)-Ia* or *aac(6’)-Ii* genes. The *int*-*Tn* gene, encoding the integrase of the Tn916-Tn1545 family of conjugative transposons was detected in three *L. saliavrius* isolates (22a, 28a, 30a). Its presence was in coexistence with *tetM* and *tetL*, and the two isolates also contained the *ermB* gene.

### Nitrocefin test

The results of the nitrocefin test for the rapid chromogenic detection of beta-lactamase activity [24] was negative for all *Lactobacillus* isolates phenotypically resistant to ampicillin.

## Discussion

In this paper we have presented the first report on the identification and antibiotic susceptibility of lactobacilli from farm turkeys.

Bacteria were identified to the species level using MALDI-TOF MS and, if uncertain results were obtained, identification was further based on 16S rDNA analysis. The reliability and effectiveness of MALDI-TOF MS in typing lactobacilli has been confirmed by several authors and high agreement has been observed between results obtained in mass spectrometry and in various genetic methods, even if the log(score) values were lower than 2.00 [13, 25, 26]. However, in this work we found that MALDI-TOF MS had insufficient discriminatory power to differentiate closely related species such as *L. johnsonii* and *L. gasseri*, *L. crispatus* and *L. ultunensis*, and *L. oris* and *L. antri.* Homology between *L. johnsonii* and *L. gasseri* at the sequence level of 16S rDNA and other genes is known to be very high [27], and the similarity of the 16S rDNA sequence between *L. crispatus* and *L. ultunensis* and between *L. oris* and *L. antri* has been estimated at 98.2% and 99.8%, respectively [28]. The results of our work showed that the genetic similarity between these species translates into similarity in cellular protein profiles. However, as shown by restriction analysis of 16S rDNA, despite high homology between mass spectra of closely related species, the first best match (log(score)≥1.70) was correct for all questionable samples. The issue of ambiguous differentiation of closely related *Lactobacillus* species in MALDI-TOF MS, including *L. johnsonii* and *L. gasseri*, has been addressed also in our earlier paper [13].

The *Lactobacillus* species identified in this study in isolates from turkeys are similar to those found in poultry worldwide. Several reports have pointed out the predominance of *Lactobacillus crispatus*, *L. salivarius*, *L. reuteri and L. johnsonii* among intestinal autochthonic chicken lactobacilli [29, 30]. In Poland, the dominant *Lactobacillus* species in geese and chickens are *L. salivarius* and *L. johnsonii*, and the remaining species which have been identified in turkeys, i.e. *L. crispatus*, *L. ingluviei*, *L. reuteri*, *L. oris*, *L. agilis* and *L. saerimneri*, were isolated with a lower frequency [25, 31].

Among the lactobacilli tested we found high prevalence of resistance to tetracycline (68% resistant isolates), lincomycin (64.5%), enrofloxacin (60%) and ampicillin (50%). The frequency of resistance to other amicrobial agents, i.e. erythromycin, aminoglycosides and chloramphenicol, ranged from 10% to 45%. The high level of antibiotic resistance observed in this study is probably due to the widespread use of antimicrobial drugs on turkey farms. The history of the use of antibiotics in the flocks from which *Lactobacillus* isolates were derived was not made available. However, according to the inspection carried out in Poland in 2015-2016 by the Supreme Audit Office, antibiotics were detected in 88% of turkey farms (in water or feed). The standards for doxycycline and enrofloxacin were exceeded most often, and antibiotics most commonly used in Poland in animal husbandry include tetracyclines and penicillins [32]. These facts may justify the high prevalence of resistance to ampicillin, tetracycline and enrofloxacin in the tested lactobacilli.

High prevalence of tetracycline resistance (68% of isolates) in turkey lactobacilli is in line with our earlier research demonstrating that 75% of *Lactobacillus* isolates from chickens in Poland are resistant to this antibiotic [16]. Similar results obtained also Cauwerts et al. [33], who recorded nearly 80% resistance to tetracycline among lactobacilli from Belgian broiler farms. The observed tetracycline resistance was due to the presence of *tet* genes, which code for energy-dependent efflux proteins (*tetL*) or for a protein that protects bacterial ribosomes from the action of tetracyclines (*tetM*, *tetW*) [34]. The incidence of *tetL, tetM* and *tetW* genes in turkey lactobacilli is similar to that occurring in *Lactobacillus* isolates from chicken farms in Poland and Belgium [16, 33]. The *tetW* and *tetM* genes are also widespread in *Lactobacillus* bacteria isolated from humans and food products [35]. Our finding that the *tetW* gene is characteristic for the isolates belonging to the phylogenetic group *L. delbrueckii* and *L. reuteri*, and that its occurrence among isolates of the *L. salivarius* group is sporadic, is consistent with previous research on chicken lactobacilli [16].

The high rates of resistance (45-63%) to MLS antibiotics (macrolides, lincosamides and streptogramins) observed in turkey lactobacilli are in line with our recent study showing that 70% of lactobacilli derived from chickens in Poland were resistant to lincomycin and 42% were resistant to macrolides [16]. Similar findings were reported by Cauwerts et al. [19] for *Lactobacillus* bacteria isolated from broiler chicken in Belgium. The bimodal distribution of MIC values for erythromycin, which suggests acquired resistance, has been also recorded earlier for lactobacilli of various origins [19, 36].

Phenotypic resistance to MLS antibiotics in the turkey lactobacilli was associated with the presence of *erm* genes, which encode rRNA methylases, and the *lnuA* gene, which encodes lincosamide *O*-nucleotidyltransferase. The high incidence of the *ermB* gene (in 45% of isolates) and the lower incidence of *ermC* (6%) is consistent with previous studies on the antibiotic susceptibility of chicken *Lactobacillus* strains [16, 19]. In contrast, the frequency of the *lnuA* gene in turkey lactobacilli (18%) was about half of that noted in chicken isolates (39%) in Poland, although phenotypic resistance to lincomycin was very similar in both species [16]. The presence of the *ermC* gene not only in resistant but also MLS-suseptible *Lactobacillus* isolates has also previously been reported by other authors [16, 25].

The incidence of ampicillin resistance (47% resistant isolates) recorded in this study is much higher than that observed by other researchers working on poultry LAB [37, 38]. The ampicillin resistance rate in chicken lactobacilli (26%) from Polish farms is almost half that of turkey *Lactobacillus* isolates [16]. High MIC values for ampicillin (≥64 μg/ml) recorded for 24% isolates were usually accompanied by elevated MICs for first, second and thirs generation cephalosporins (≥16 μg/ml) indicating cross-resistance. The mechanism of resistance of the lactobacilli to ampicillin remained unexplained, but the results of the tests carried out excluded the involvement of β-lactamases. This is line with studies by other authors [23, 39], who have demonstrated the absence of the *bla*Z gene encoding β-lactamase in lactobacilli phenotypically resistant to penicillins. The third generation cephalosporins are usually highly resistant to βlactamases.

Among turkey lactobacilli, 30% of isolates showed resistance to chloramphenicol, and for most of them (12 of 18 phenotypically resistant) the MIC was 8 μg/ml, while the EFSA threshold is 4 μg/ml. Higher MIC values, i.e. 16-128 μg/ml, and the presence of the chloramphenicol acetyltransferase *cat* gene were characteristic only for *L. ingluviei* isolates, although two cat-positive strains were considered phenotypically susceptible. A similar range of MIC values for chloramphenicol, i.e. 1-8 μg/ml for most lactobacilli tested, has been observed by other authors [16, 39, 40], while high MIC values ≥32 μg/ml have been noted only occasionally [14, 16, 40]. The presence of the *cat* gene among chloramphenicol-susceptible *Lactobacillus* isolates has also been observed in our previous work, and more precise studies by Hummel et al. [23] showed that the *cat* gene was not expressed (RNA level) in some *cat*-positive but phenotypically susceptible LAB strains.

In this work, we observed a fairly high frequency of resistance to streptomycin (31%), while gentamicin resistance was much less prevalent (10%). A similar percentage (12.5-31%) of aminoglycoside-resistant strains was recorded for chicken lactobacilli in Poland [16]. More frequent occurrence of resistance to streptomycin than to gentamicin among lactobacilli from various sources was also demonstrated by Danielsen and Wind [40]. Of the genes that determine resistance to aminoglycosides, only two are found in, ie. *aadE* and *ant(6)-Ia*, in 3 isolates of *L. salivarius* showing phenotypic resistance to streptomycin. According to Ramires and Tolmasky [41], *aadE* and *ant(6)-Ia* encode O-adenyltransferases that confer resistance to streptomycin and belong to the ANT(6) group of modifying enzymes but the results of sequence analysis of PCR products indicated that *aadE* and *ant(6)-Ia* are the same gene detected by different primers. The *aadE* or *ant(6)-Ia* gene has been previously detected in *L. salivarius* strains from chicken [16] and in *L. casei* and *L. plantarum* isolates from food sources or human biopsy samples [42].

The present study provides the second report on the sensitivity of lactobacilli to tiamulin. In our previous work on chicken lactobacilli, we proposed a concentration of 8 μg/ml as a breakpoint for distinguishing sensitive and resistant strains. In the present study we have not adopted this cut-off point, nor have we proposed other breakpoint values, due to the MIC distribution and insufficient number of isolates of most *Lactobacillus* species. However, the high MIC values of tiamulin, i.e. ≥32 μg/ml, noted in the majority (61%) of turkey lactobacilli suggest the prevalence of resistance to this antibiotic. As in the earlier studies on chicken lactobacilli [16] only 10% isolates were tiamulin MIC values as low as ≤0.5 μg/ml. The genetic resistance of most *Lactobacillus* strains with high MIC values for tiamulin has not been determined. The *lsaE* gene that codes for ATP-dependent drug efflux pump was detected only in 3 *L. salivarius* isolates with high MIC values (128-256 μg/ml) of tiamulin. Therefore, it is likely that the low sensitivity of the lactobacilli to this pleuromutulin is the modification of the target, i.e. 23S rRNA at the peptidyl transferase center of the 50S subunit [43]. All *lasE*-positive isolates simultaneously contained the *aadE*/*ant(6)-Ia* gene conferring resistance to streptomycin. This observation is in line with our recent findings on chicken lactobacilli [16] and previous reports describing the occurrence of *lsaE* within plasmid or chromosomal clusters comprising several resistance genes, including *aadE* [44].

The percentage of enrofloxacin-resistant *Lactobacillus* strains (MIC≥64 μg/ml) in the turkeys (60%) was higher than in chickens (48%) and in geese (23%) in Poland [16, 45]. Lactobacilli from other sources, such as dairy products or cattle intestine, are usually sensitive to enrofloxacin [46, 47].

The Tn*916*/Tn*1545*-like coniugative transposon that was identified in three strains of *L. salivarius*, is commonly found in various bacteria, including entrococci and streptococci, but not in lactobacilli [48, 49]. The coexistence of integrase gene *int-tn* and *tetM*, *tetL* and *ermB* genes observed in these studies is consistent with the literature data, according to which members of the Tn*916*-Tn*1545* family carry the tetracycline-resistance determinant *tetM*, as well as additional resistance genes [49].

## Conclusions

Our work is the first report on the identification and antibiotic susceptibility of *Lactobacillus* bacteria from turkeys. We have shown the predominance of *L. salivarius* (35%) and *L. crispatus* (21%) among turkey lactobacilli and a high frequency of resistance (≥45% resistant isolates) to tetracycline, lincomycin, ampicillin and erythromycin. These data indicate that antibiotic resistance has reached a dangerous level in the commensal microflora, and the high rate of ampicillin resistance thus far observed in lactobacilli is particularly alarming. There is need to promote the rational use of antibiotics in poultry farming to limit the development of resistance in bacteria. More emphasis should be placed on alternative therapies and the implementation of biosecurity practices, which are the most effective, cheapest and safest way to prevent the spread of disease on farms. Consideration should also be given to amending legislation governing the use of antibiotics in livestock. The level of antibiotic resistance may be reduced by introducing an obligation to report to the regulatory authorities the use of antibiotics on farm and by requiring antibiotic resistance tests before antibiotic use.

Our studies have shown that the natural intestinal microflora of turkeys is a reservoir of resistance genes. Many of them were previously found in LAB on the mobile elements, which can be readily transferred to other bacteria inhabiting the intestine of the host and spread in the environment [9, 48]. Further research is needed to clarify the mechanism of low sensitivity of lactobacilli to beta-lactam antibiotics and pleuromutilins.

## Additional files

Additional file 1: **Table S1.** Containing the results of sequencing PCR products (for representative wild-type isolates) that are counterparts of resistance genes and results of comparative analysis of the obtained sequences with the reference sequences deposited at GenBank. (DOC 155 kb)

Additional file 2: **Table S2.** Containing original data on the identification of bacteria by MALDI-TOF MS, MIC values and the occurrence of resistance genes. (XLS 75 kb)

Additional file 3: **Table S3.** Containing sizes (bp) of restriction fragments obtained by cleavage of 16S rDNA amplicons of reference and wild-type isolates of *Lactobacillus*. (DOC 31 kb)

## Abbreviations

16S-ARDRA: Amplified Ribosomal DNA Restriction Analysis of 16S rDNA
dNTPs: Deoxynucleoside triphosphates
EFSA’s FEEDAP Panel: European Food Safety Authority Panel on Additives and Products or Substances used in Animal Feed
G+C: Guanine + cytosine
GIT: Gastrointestinal tract
LAB: Lactic acid bacteria
LSM: LAB susceptibility test medium
MALDI-TOF MS: Matrix assisted laser desorption ionization-time of flight mass spectrometry
MIC: Minimal inhibitory concentration
MLS: Macrolides, lincosamides and streptogramins
MRS: Man, Rogosa and Sharp
PCR: Polymerase chain reaction
rDNA: Ribosomal deoxyribonucleic acid
rRNA: Ribosomal ribonucleic acid
