# Supplementary material for: Identification and antibiotic susceptibility of lactobacilli isolated from turkeys
Source: BMC Microbiol. 2018 Oct 29;18:168. doi: 10.1186/s12866-018-1269-6 (PMC6206647; doi:10.1186/s12866-018-1269-6)
Supplement: Supplementary file 1 — Table S1. Containing the results of sequencing PCR products (for representative wild-type isolates) that are counterparts of resistance genes and results of comparative analysis of the obtained sequences with the reference sequences deposited at GenBank. (DOC 155 kb) [file 12866_2018_1269_MOESM1_ESM.doc]

**Additional file 1. Sequences of PCR products being counterparts of resistance genes (control strains) and results of comparative analysis of the obtained sequences with reference sequences from the GenBank database.**

| **Gene** | **Strain** | **Amplicon sequence** | **Results of comapartive analysis using NCBI BLAST algorithm** | | | |
| --- | --- | --- | --- | --- | --- | --- |
| **Description** | **Query cover [%]** | **Identity [%]** | **Accession number GeneBank** |
| ***tetL*** | *L. salivarius* 3aI | AGACWTCTTCTACTMTTCCTATGATAACAATTATCACTGTTCCGTTTCTTATGAAATTATTAAAGAAAGAAGTAAGGATAAAAGGTCATTTTGATATCAAAGGAATTATACTAATGTCTGTAGGCATTGTATTTTTTATGTTGTTTACAACATCATATAGCATTTCTTTTCTTATCGTTAGCGTGCTGTCATTCCKAA | [Streptococcus agalactiae pMV158 tet(L) gene for tetracycline efflux MFS transporter Tet(L), complete CDS](https://blast.ncbi.nlm.nih.gov/Blast.cgi#alnHdr_1035502356) | 95 | 99 | [NG_048207.1](https://www.ncbi.nlm.nih.gov/nucleotide/NG_048207.1?report=genbank&log$=nucltop&blast_rank=39&RID=B84DKJW101N) |
| [Enterococcus faecium tet(L) gene for tetracycline efflux MFS transporter Tet(L), complete CDS](https://blast.ncbi.nlm.nih.gov/Blast.cgi#alnHdr_1035502355) | 95 | 99 | [NG_048206.1](https://www.ncbi.nlm.nih.gov/nucleotide/NG_048206.1?report=genbank&log$=nucltop&blast_rank=40&RID=B84DKJW101N) |
| ***tetM*** | *L. salivarius* 3aI | GCATCGCTTTTAGACGTMGAGAGGAATTACAATTCAGACAGGAATAACCTCTTTTCAGTGGGAAAATACGAAGGTGAACATCATAGACACGCCAGGACATATGGATTTCTTAGCARAAGTATATCGTTCATTATCAGTTTTAGATGGGGCAATTCTACTGATTTCTGCAAAAGATGGCGTACAAGCACAAACTCGTATATTATTTCATGCACTTAGGAAAATGGGGATTCCCACAATCTTTTTTATCAATAARATTGACCAAAATGGAATTGATTTATCAACGGTTTATCAGGATATTAAAGAGAAACTTTCTGCCGAAATTGTAATCAAACARAAGGTARAACTGTATCCTAATATGTGTGTGACRAACTTTACSGA | [Streptococcus pneumoniae 9409 tet(M) gene for tetracycline resistance ribosomal protection protein Tet(M), complete CDS](https://blast.ncbi.nlm.nih.gov/Blast.cgi#alnHdr_1035502402) | 98 | 98 | [NG_048253.1](https://www.ncbi.nlm.nih.gov/nucleotide/NG_048253.1?report=genbank&log$=nucltop&blast_rank=86&RID=B84R67P201N) |
| [Staphylococcus aureus 4520 tet(M) gene for tetracycline resistance ribosomal protection protein Tet(M), complete CDS](https://blast.ncbi.nlm.nih.gov/Blast.cgi#alnHdr_1035502398) | 98 | 98 | [NG_048249.1](https://www.ncbi.nlm.nih.gov/nucleotide/NG_048249.1?report=genbank&log$=nucltop&blast_rank=89&RID=B84R67P201N) |
| [Lactobacillus plantarum 5057 pMD5057 tet(M) gene for tetracycline resistance ribosomal protection protein Tet(M), complete CDS](https://blast.ncbi.nlm.nih.gov/Blast.cgi#alnHdr_1035502374) | 98 | 98 | [NG_048225.1](https://www.ncbi.nlm.nih.gov/nucleotide/NG_048225.1?report=genbank&log$=nucltop&blast_rank=97&RID=B84R67P201N) |
| ***tetO*** | *E. faecalis* 140 | GGGAGGAMGMTTARCGGAAAGTTTATTGTATACCAGTGGTGCAATTGCAGAACTAGGGAGCGTAGATGAAGGCACAACAAGGACAGATACAATGAATTTGGAGCGTCAAAGGGGAATCACTATCCAGACAGCAGTGACATCTTTTCAGTGGGAGGATGTAAAAGTCAACATTATAGATACGCCAGGCCATATGGATTTTTTGGCGGAAGTATACCGTTCTTTATCCGTATTAGACGGAGCAGTATTATTAGTTTCTGCAAAGGATGGCATACAGGCACAGACCCGTATACTGTTTCATGCACTACAGATAATGAAGATTCCGACAATTTTTTTCATCAATAAAATTGACCAAGAGGGGATTGATTTGCCAATGGTATATCGGGAAATGAAAGCAAAGCTTTCTTCGGAAATTATAGTGAAGCAAAAGGTTGGGCAGCATCCCCATATAAATGTAACGGACAATGACGATATGG | [Bifidobacterium longum strain Y1 TetO (tetO) gene, complete cds; and Cpp-like gene, complete sequence](https://blast.ncbi.nlm.nih.gov/Blast.cgi#alnHdr_1231295767) | 97 | 99 | [KY697303.1](https://www.ncbi.nlm.nih.gov/nucleotide/KY697303.1?report=genbank&log$=nucltop&blast_rank=7&RID=B850RS0Z014) |
| [Campylobacter jejuni tet(O) gene for tetracycline resistance ribosomal protection protein Tet(O), complete CDS](https://blast.ncbi.nlm.nih.gov/Blast.cgi#alnHdr_1035502406) | 97 | 99 | [NG_048257.1](https://www.ncbi.nlm.nih.gov/nucleotide/NG_048257.1?report=genbank&log$=nucltop&blast_rank=45&RID=B850RS0Z014) |
| [Streptococcus equi subsp. zooepidemicus tetO gene for tetracycline resistance protein TetO, partial cds, isolate: Strep243](https://blast.ncbi.nlm.nih.gov/Blast.cgi#alnHdr_1004530861) | 97 | 99 | [LC131137.1](https://www.ncbi.nlm.nih.gov/nucleotide/LC131137.1?report=genbank&log$=nucltop&blast_rank=73&RID=B850RS0Z014) |
| ***tetW*** | *L. salivarius* 27eCh | AKCGKTCTGGTAGGAGTGACTGCCGCTTGAACGGTAATCCCACGCTGCCGCTCCAAAAACATGGTGTCCGTCCTCGTTGTCCCTTTTTCGACGCTCCCCGGTTCTGAAATGGCTCCGCTGGCATATAGCAGGCTCWCA | [Uncultured organism clone GW-14-2 tetracycline resistance (tetW) gene, partial cds](https://blast.ncbi.nlm.nih.gov/Blast.cgi#alnHdr_84689075) | 94 | 98 | [DQ309605.1](https://www.ncbi.nlm.nih.gov/nucleotide/DQ309605.1?report=genbank&log$=nucltop&blast_rank=1&RID=B859N7N4015) |
| [Bifidobacterium bifidum L22 tet(W) gene for tetracycline resistance ribosomal protection protein Tet(W), complete CDS](https://blast.ncbi.nlm.nih.gov/Blast.cgi#alnHdr_1035502448) | 93 | 98 | [NG_048301.1](https://www.ncbi.nlm.nih.gov/nucleotide/NG_048301.1?report=genbank&log$=nucltop&blast_rank=50&RID=B859N7N4015) |
| [Streptococcus suis 33421 tet(W) gene for tetracycline resistance ribosomal protection protein Tet(W), complete CDS](https://blast.ncbi.nlm.nih.gov/Blast.cgi#alnHdr_1035502449) | 93 | 98 | [NG_048303.1](https://www.ncbi.nlm.nih.gov/nucleotide/NG_048303.1?report=genbank&log$=nucltop&blast_rank=60&RID=B859N7N4015) |
| ***ermB*** | *L. salivarius* 3aI | TCCGCTTWWCTTTTGGTTTAGGATGAAAGCATTCCGCTGGCAGCTTAAGCAATTGCTGAATCGAGACTTGAGTGTGCAAGAGCAACCCTAGTGTTCGGTGAATATCCAAGGTACGCTTGTAGAATCCTTCTTCAACAATCAGATAGATGTCARACGCACGGCTTTCAAAAACCACTTTTTTAATAATTTGTGTGCTTAAATGGTAAGGAATATTCCCAACAATTTTATACCTCTGTTTGTTAGGGAATTGAAACTGTARAATATCTTGGTGAATTAAAGTGACACGAGTATTCAGTTTTAATTTTTCTGACGATAAGTTGAATAGATGACTGTCTAATTCAATAGACGTTACCTGTTTACTTATTTTAGCCAGTTTCGTCGTTAAATGCCCTTTACCTGTTCCAATTTCGTAAACGGTATCGGTTTCTTTTAAATTCAATTGTTTTATTATTTGGTTGAGTACTTTTTCACTCGTTAAAAAGTTTTGARAATATTTTATATTTTTGTTCATGTAATCACTCCTGAAGTGATTACATCTGTAAATAAATACARAAGTTAAACGATTTGTTTGTAATTTTAGTTATCTGTTTAAAAAGTCATAARATTAGTCACTGGTAGGAATTAATCTAACGTATTTATTTATCTGCGTAATCACTGTTTTTAGTCTGTTTCAAAACAGTARATGTTTTATCTACATTACGCATTKGGGAAAWWMCCAA | [Streptococcus pneumoniae isolate PW1981 adenine-N6 methyltransferase (ermB) gene, partial cds](https://blast.ncbi.nlm.nih.gov/Blast.cgi#alnHdr_113196895) | 97 | 99 | [DQ855647.1](https://www.ncbi.nlm.nih.gov/nucleotide/DQ855647.1?report=genbank&log$=nucltop&blast_rank=5&RID=B85HB207015) |
| [Lactobacillus fermentum plasmid conferring erythromycin resistance: rRNA methylase regulatory protein gene, rRNA methylase (erm) gene, replication protein (rep) gene, complete cds, and DNA transposase (tse) gene, partial cds](https://blast.ncbi.nlm.nih.gov/Blast.cgi#alnHdr_1230604) | 97 | 99 | [U48430.1](https://www.ncbi.nlm.nih.gov/nucleotide/U48430.1?report=genbank&log$=nucltop&blast_rank=9&RID=B85HB207015) |
| [Streptococcus pneumoniae erm(B) gene for rRNA adenine N-6-methyltransferase, strain K-65](https://blast.ncbi.nlm.nih.gov/Blast.cgi#alnHdr_67511467) | 97 | 99 | [AJ972604.1](https://www.ncbi.nlm.nih.gov/nucleotide/AJ972604.1?report=genbank&log$=nucltop&blast_rank=29&RID=B85HB207015) |
| ***ermC*** | *L. salivarius* 3aI | AGCATTGATACAAGCGCTMTTGGCGTTACTTTTAAKGGCAGAAGTTGATATTTCTATATTAAGTATGGTTCCAAGAGAATATTTTCATCCTAAACCTAAAGTGAATAGCTCGCTTATCAGATTAAATAGAAAAAAATCAAGAATATCGTACAAAGATAAACAGAAGTATAATTATTTCGTTATGAAATGGGTTAACAAAGAATACAAGAAAATATTTACAAAGAATCAATTTAACAAATCCTTAAAACATGCAGGAATTGACGATA | [Staphylococcus hyicus 46 pSES21 erm(C) gene for 23S rRNA (adenine(2058)-N(6))-methyltransferase Erm(C), complete CDS](https://blast.ncbi.nlm.nih.gov/Blast.cgi#alnHdr_1035502000) | 98 | 98 | [NG_047814.1](https://www.ncbi.nlm.nih.gov/nucleotide/NG_047814.1?report=genbank&log$=nucltop&blast_rank=2&RID=BD3YDPCF014) |
| [Lactobacillus reuteri PA-16 unnamed erm(C) gene for 23S rRNA (adenine(2058)-N(6))-methyltransferase Erm(C), complete CDS](https://blast.ncbi.nlm.nih.gov/Blast.cgi#alnHdr_1035502003) | 98 | 97 | [NG_047817.1](https://www.ncbi.nlm.nih.gov/nucleotide/NG_047817.1?report=genbank&log$=nucltop&blast_rank=15&RID=BD3YDPCF014) |
| ***lnuA*** | *L. ingluviei* 22eI | CCGCAAGAGAMCAGAGATATAGATATAGATTTTGACGCTCAACACACTCAAAAAGTTATACAAAAATTAGAARATATCGGATACAAAATARAAGTTGATTGGATGCCTTCACGTATGGAACTCAAGCATGAARAATATGGGTATTTARATATTCATCCTATAAATCTAAATGATGATGGATCAATTACCCAAGCAAACCCARAAGGTGGTAATTATGTTTTCCAAAATGACTGGTTTTCARAAACTAATTACAAARATCGAAAAATACCATGTATTTCAAAARAASMA | [Staphylococcus epidermidis pLNU6 (naturally occurring) lnu(A) gene for lincosamide nucleotidyltransferase Lnu(A), complete CDS](https://blast.ncbi.nlm.nih.gov/Blast.cgi#alnHdr_1035502101) | 95 | 96 | [NG_047919.1](https://www.ncbi.nlm.nih.gov/nucleotide/NG_047919.1?report=genbank&log$=nucltop&blast_rank=5&RID=B85WN9M8014) |
| [Staphylococcus haemolyticus lnu(A) gene for lincosamide nucleotidyltransferase Lnu(A), complete CDS](https://blast.ncbi.nlm.nih.gov/Blast.cgi#alnHdr_1035502095) | 95 | 96 | [NG_047913.1](https://www.ncbi.nlm.nih.gov/nucleotide/NG_047913.1?report=genbank&log$=nucltop&blast_rank=22&RID=B85WN9M8014) |
| ***aph(2’)-Ic***  ***(aph(2’)-IIIa)*** | *L. salivarius 27eCh* | TTTTCGGTTTGTAMGGAACCCCGGCTGATATGGCAGTTTCAACAGGAAAKGCGCTTAGCTCATTCATGAACTCAGCAAGTTGCAGCGCCAGTCGATCTTTTGCATCATCGGGAAAAACGGCCATCCCGTCTTCMCCCAAGATTTGGCCTTGGACTTTACGGTAGCCCACGAAGGGATTTCCATCACTTCGCTTTCCRATATATACATACKGTGGAATATTCACCTTAACACAACCGACCAACAGAGGTAGCAATTGGATTTCTTTGTTCAATTCGTCTGCACCTTGTTGACTCTTGGRAAAACRAAAAACCCAATCTCCATTGACRAGGATCGCGTAATTCCTAAAGCCCTCACCAAGCGACTCGACGGATTGTATGCTTATATCTGGGAACTGAGTCAT  TATCATTGKGG | [Campylobacter coli strain ZTA14/01426 aminoglycoside 2-phosphotransferase IIIa (aph(2)-IIIa), 3-aminoglycoside o-phosphotransferase type IIIa (aph(3)-IIIa), phosphorylase (pnp), spectinomyicin adenyltransferase (aad9), erythromycin resistance methylase B (erm(B)), and Aad6 (aad6) genes, complete cds](https://blast.ncbi.nlm.nih.gov/Blast.cgi#alnHdr_1293068907) | 100 | 97 | [MF134832.1](https://www.ncbi.nlm.nih.gov/nucleotide/MF134832.1?report=genbank&log$=nucltop&blast_rank=1&RID=B8644HCR014) |
| [Enterococcus gallinarum SF9117 aph(2'')-IIIa gene for aminoglycoside O-phosphotransferase APH(2'')-IIIa, complete CDS](https://blast.ncbi.nlm.nih.gov/Blast.cgi#alnHdr_1035501628) | 100 | 97 | [NG_047399.1](https://www.ncbi.nlm.nih.gov/nucleotide/NG_047399.1?report=genbank&log$=nucltop&blast_rank=2&RID=B8644HCR014) |
| [Enterococcus gallinarum gentamicin resistance protein gene, complete cds](https://blast.ncbi.nlm.nih.gov/Blast.cgi#alnHdr_1854638) | 100 | 97 | [U51479.1](https://www.ncbi.nlm.nih.gov/nucleotide/U51479.1?report=genbank&log$=nucltop&blast_rank=3&RID=B8644HCR014) |
| ***aph(2’)-Id***  ***(aph(2’)-IVa)*** | *E. faecium* 60 | TGGTCGCAGATATCTTTTCGAACGACCAGTATTTTTCTTTCATCCTATATTTTTCCAAAACTGTCGGTATATCCTTATGTTTGTAATGGTTCAATATTTTTGATACAAATTCCATGCCGTATTCTTCATCATCTTCCATCAAACTTATAAAATCATTGTCGGGATCARAAATAGCTGCATCTCCAAAATCGATTATTCCACAAATAGTATTTTTTTCGGTATCAAATAAAATATGATCACTGCTAAAATCGTTATGAATAARACAAGGATAGTATTTGAAGTARATTTCGTTCTCTARAATATCCCTGTAAAAATCATCCACTTTCTTCATCTGGGGACCCTTTAATTCCCTGGATAGTAACTTTTTGATTTTTTTATTATCTTCATTTATCTTCTCTCGAAAATCTAATACCARATTACTTTTGAATCCARAGATGTTTATGCTGTGAAGTTCACTTARAAATCGGGCCAGGTCCTTAGCTGCCTGATTTTGAGATTGCTTCGGCARATTATTGAGTARAARAGGTGTCAATGGTACTCCTTTAATTTTTGTAAAACCTGCRAAAGACATTTGGTACGTTTCTGATGGCATTCCTGAAAAAAAMCMMC | [Enterococcus casseliflavus aminoglycoside modifying enzyme (aph(2'')-Id) gene, complete cds](https://blast.ncbi.nlm.nih.gov/Blast.cgi#alnHdr_3080754) | 97 | 98 | [AF016483.1](https://www.ncbi.nlm.nih.gov/nucleotide/AF016483.1?report=genbank&log$=nucltop&blast_rank=3&RID=B86CSN7F014) |
| [Enterococcus faecium APH(2'')-Id (aph(2'')-Id) gene, complete cds](https://blast.ncbi.nlm.nih.gov/Blast.cgi#alnHdr_57918601) | 97 | 95 | [AY743255.1](https://www.ncbi.nlm.nih.gov/nucleotide/AY743255.1?report=genbank&log$=nucltop&blast_rank=7&RID=B86CSN7F014) |
| ***ant(4’)-Ia*** | *E. faecalis* 3 | GGASATGGTMTAACCTGAAGGAAGATCTGATTGCTTAACTGCTTCAGTTAARACCGAAGCGCTCGTCGTATAACARATGCRATGATGCARACCAATCAACATGGCACCTGCCATTGCTACCTGTACAGTCAAGGATGGWARAAATGTTGTCGGYCCTTGCACACRAATATTACSCCWTTTGCCTGCATATTCAAACAGCTCTTCTACRATAAGGGCACAAATCGCATCGKGRAACGTTTGGSCTTCTACCGATTWAGCAGTTTAAAAATATGCAGGCAAATGGCGTAATAWTCGTGKCMAGACGACAACATTTCTACCATCCTTGAGTGTACAGGTAGCRWTGGCAGGTGCCATGTTGATTGGTCTGCATCATCSCAYCTYTTATACAAGAGCGCTTCGTTCTTAACTRAAGCATTARGCATTCARAWCTTCCTTCMTGTTATGACCTCTGTGCCGTTCWAAWACTGGTCAWCTTTCCCM | [Staphylococcus sciuri strain wo48-2 plasmid pWo48-2 aminoglycoside acetyltransferase/aminoglycoside phosphotransferase (aacA-aphD), transposase (tnpF), kanamycin nucleotidyltransferase (aadD)](https://blast.ncbi.nlm.nih.gov/Blast.cgi#alnHdr_1160547883)**,** bleomycin resistance protein (ble), IstA (istA), IstB (istB), 23S rRNA methylase (cfr), Res (res), and transposase (tnp) genes, complete cds | 98 | 93 | [KX982175.1](https://www.ncbi.nlm.nih.gov/nucleotide/KX982175.1?report=genbank&log$=nucltop&blast_rank=11&RID=B88075TY014) |
| [Staphylococcus aureus HUC19 pUB110 aadD1 gene for aminoglycoside O-nucleotidyltransferase ANT(4')-Ia, complete CDS](https://blast.ncbi.nlm.nih.gov/Blast.cgi#alnHdr_1035501610) | 98 | 93 | [NG_047375.1](https://www.ncbi.nlm.nih.gov/nucleotide/NG_047375.1?report=genbank&log$=nucltop&blast_rank=33&RID=B88075TY014) |
| ***aph(3’)-IIIa*** | *E. faecalis* 3 | TGGGAGTGTCTTCTTCCMGTTTTCGCAATCCACATCGGCCAGATCGTTATTCAGTAAGTAATCCAATTCGGCTAAGCGGCTGTCTAAGCTATTCGTATAGGGACAATCCGATATGTCGATGGAGTGAAAGAGCCTGATGCACTCCGCATACAGCTCGATAATCTTTTCAGGGCTTTGTTCATCTTCATACTCTTCCRAGCAAAGGACGCCATCGGCCTCACTCATGAGCAGATTGCTCCAGCCATCATGCCGTTCAAAGKGCAGGACCTTTGGAACAGGCAGCTTTCCTTCCAGCCATAGCATCATGTCCTTTTCCCGTTCCACATCATAGGTGGTCCCTTTATACCGGCTGTCCGTCATTTTTAAATATAGGTTTTCATTTTCTCCCACCAGCTTATATACCTTAGCAGGAGACATTCCTTCCGTATCTTTTACGCAGCGGTATTTTTCGATCAKTTTTTTCAATTCCGGTGATATTCT | [Campylobacter coli strain ZTA14/01426 aminoglycoside 2-phosphotransferase IIIa (aph(2)-IIIa), 3-aminoglycoside o-phosphotransferase type IIIa (aph(3)-IIIa), phosphorylase (pnp), spectinomyicin adenyltransferase (aad9), erythromycin resistance methylase B (erm(B)), and Aad6 (aad6) genes, complete cds](https://blast.ncbi.nlm.nih.gov/Blast.cgi#alnHdr_1293068907) | 99 | 99 | [MF134832.1](https://www.ncbi.nlm.nih.gov/nucleotide/MF134832.1?report=genbank&log$=nucltop&blast_rank=28&RID=B86J9MBJ015) |
| [Staphylococcus haemolyticus strain 1404 aminoglycoside 3'-phosphotransferase (aphA) gene, complete cds](https://blast.ncbi.nlm.nih.gov/Blast.cgi#alnHdr_1261491472) | 99 | 99 | [KY100260.1](https://www.ncbi.nlm.nih.gov/nucleotide/KY100260.1?report=genbank&log$=nucltop&blast_rank=38&RID=B86J9MBJ015) |
| ***aac(6’)-Ie-aph(2”)-Ia*** | *E. faecalis* 3 | ACTTCTAAACMGATTTTTGGTATGCCCTTATTGCTCTTGGATTATTTTTATGAGGGTCTAAAATAACTGCATTAGCATTTCTTTCTTTTTTCAAAAATTCAAAAATCAATTTAATATATCTTGTACCAATTCCTTTACTCCAATAATTTGGCTCTCCTATAAATTGATCCATACCATAGACTATCTCATCAGTTTTTGGATAATGATAATCAGTATATAACTCATCATACATTTTATATATTTGTCCATATCCAATAGGAACATTGTTATATTCAATAATTACTCTAAAAACTTCATCTTCCCAAGGCTCKAA | [Staphylococcus aureus hypothetical protein gene, partial cds; and cassette chromosome recombinase CcrC2 (ccrC2), bifunctional aminoglycoside N-acetyltransferase AAC(6')-Ie/aminoglycoside O-phosphotransferase APH(2''), IS431 transposase, mecA-type methicillin resistance repressor (mecI), signal transducer protein (mecR1), MecA (mecA), and IS431 transposase genes, complete cds](https://blast.ncbi.nlm.nih.gov/Blast.cgi#alnHdr_1354558580) | 98 | 99 | [MG674089.1](https://www.ncbi.nlm.nih.gov/nucleotide/MG674089.1?report=genbank&log$=nucltop&blast_rank=3&RID=B86ZKKYZ014) |
| [Staphylococcus sciuri strain wo48-2 plasmid pWo48-2 aminoglycoside acetyltransferase/aminoglycoside phosphotransferase (aacA-aphD), transposase (tnpF), kanamycin nucleotidyltransferase (aadD), bleomycin resistance protein (ble), IstA (istA), IstB (istB), 23S rRNA methylase (cfr), Res (res), and transposase (tnp) genes, complete cds](https://blast.ncbi.nlm.nih.gov/Blast.cgi#alnHdr_1160547883) | 98 | 99 | [KX982175.1](https://www.ncbi.nlm.nih.gov/nucleotide/KX982175.1?report=genbank&log$=nucltop&blast_rank=30&RID=B86ZKKYZ014) |
| [Staphylococcus aureus SK18 pSK1 gene for bifunctional aminoglycoside N-acetyltransferase AAC(6')-Ie/aminoglycoside O-phosphotransferase APH(2'')-Ia, complete CDS](https://blast.ncbi.nlm.nih.gov/Blast.cgi#alnHdr_1035501463) | 98 | 99 | [NG_047055.1](https://www.ncbi.nlm.nih.gov/nucleotide/NG_047055.1?report=genbank&log$=nucltop&blast_rank=74&RID=B86ZKKYZ014) |
| ***aac(6’)-Ii*** | *E. faecium* 24 | TSGGCAGAGTAGAGAATGATGAATCCAGAACGAATCGCGGTAGCAGCGGTAGACCAAGATGAGTTAGTAGGATTTATTGGTGCAATCCCTCAATACGGTATCACAGGTTGGGAATTGCATCCATTAGTTGTAGAAAGCTCCCGACGAAAGAACCAAATAGGTACTCGATTAGTCAATTACTTAGAAAAAGAAGTAGCTTCCAGAGGAGGAATCACGATTTATTTAGGTACGGATGATTTAGACCATGGAACAACGTTAAGTCAAACGGACCTGTATGAGCATACATTTGATAAAGTGGCTTCTATCCAGAACCTTCGTGAACATCCGTATGAATTCTATGAAAAATTAGGTTATAAAATCGTAGGTGTCTACCCAAATGMA | [Enterococcus faecium DO aac(6') gene for aminoglycoside 6'-N-acetyltransferase, complete CDS](https://blast.ncbi.nlm.nih.gov/Blast.cgi#alnHdr_1127916826) | 97 | 99 | [NG_052371.1](https://www.ncbi.nlm.nih.gov/nucleotide/NG_052371.1?report=genbank&log$=nucltop&blast_rank=26&RID=B878BYVA015) |
| [Enterococcus faecium MRSN 4777 aac(6') gene for aminoglycoside 6'-N-acetyltransferase, complete CDS](https://blast.ncbi.nlm.nih.gov/Blast.cgi#alnHdr_1127919959) | 97 | 98 | [NG_052465.1](https://www.ncbi.nlm.nih.gov/nucleotide/NG_052465.1?report=genbank&log$=nucltop&blast_rank=55&RID=B878BYVA015) |
| ***ant(6)-Ia*** | *L. salivarius* 5aI | TTCATCTTGAGCAGTGTATTTAGATATGTGGTCAGTGTTCTATGATATTTTGTAACCTTTTCTTCCAAAAACATAGCTGTCCGTTTGCCCTCATAATAATAATTGTCCACCGGGAAATGCAGTATTTCTCCTTTTTCGTTATAATACCAGTCTTGTGTTCCATGAGCAGTAAAAACAGGATGTTCAACTGTAAAAACTAAATTGCCACCAGCCTTCAGCATCCTATATATCTTTTTTATTAAATTCTCATAGTCTGCTACATAATGAAACGCAAGCGAACTTAGTATTACATCAAAGCTCTCCTCTGGGAAATCCACATCTTCTATGGCACAGCATTCATATTCAATCTGTGGAAAATGGGTTTTTCCTTTTGCTACTTCGAGCATTTTATGAGAAATATCAACACCTACTACAGAGGAAGCACCGTTTTCCATCGCATATATACAGTGCCATCCATAGCCGCATCCTAAATCAAGCACACGCTTACCCTTAAAATCAGGTAGCATCTTTTTCAAAGTCTCCCATTTCTYCCCGA | [Streptococcus agalactiae strain SGB76 insertion sequence IS1216E transposase (tnp) gene, complete cds; and aminoglycoside 6-adenylyltransferase (aadE), putative adenine phosphoribosyltransferase (apt), putative spectinomycin adenylyltransferase (spc), hypothetical proteins, ABC transporter, lincosamide nucleotidyltransferase (lnuB), transposase-like ISL3 family protein (transposase), hypothetical protein, and putative methyltransferase protein genes, complete cds](https://blast.ncbi.nlm.nih.gov/Blast.cgi#alnHdr_576639548) | 98 | 99 | [KF772204.1](https://www.ncbi.nlm.nih.gov/nucleotide/KF772204.1?report=genbank&log$=nucltop&blast_rank=83&RID=B9KW6G6U015) |
| [Enterococcus faecalis strain E505 lsa(E)-carrying multidrug resistance gene cluster, partial sequence](https://blast.ncbi.nlm.nih.gov/Blast.cgi#alnHdr_1039262215) | 98 | 99 | [KX156279.1](https://www.ncbi.nlm.nih.gov/nucleotide/KX156279.1?report=genbank&log$=nucltop&blast_rank=58&RID=B9KW6G6U015) |
| [Staphylococcus aureus insertion sequence IS257 transposase (tnp) gene, partial cds; hypothetical protein, putative methyltransferase protein, aminoglycoside 6-adenylyltansferase (aadE), ad](https://blast.ncbi.nlm.nih.gov/Blast.cgi#alnHdr_391225697) | 98 | 99 | [JQ861959.1](https://www.ncbi.nlm.nih.gov/nucleotide/JQ861959.1?report=genbank&log$=nucltop&blast_rank=89&RID=B9KW6G6U015) |
| ***aadE*** | *L. salivarius* 5aI | GAMTCTGATCTCTATCATCATCTACAATCAAAACTCTTTTGTTCCTCACAATAGAACTCTTAGATATTTCAAAGCTTTTATCTTGACCTGAATAGTCGGTAAAATGTGTCGAAATAATCTCTTCTTTTGCGTATGGCAGCTTATCACCTTTTCTTACTCCAATAAACCCCACTCCAAGTTCTTTGGCTATGGCAGTGCCTAGTATCCATCCAATTGCTTCTGGGGCAGCCACATAATCTACTTTATTTTGAAAATCCAATGATAGTACTTTTACAATTTTATCAAAAACGGTTTTATGTGTAAAAATAGTTAGTAAATCATATTTTCCAATTGAGTTACGAGGTAATATACCTCTAATTTCATTTATAACATCAGTACTTTCCATACATTTACACCCCTTGCTTATGGTTAATAATCTTTGCTAATCTTTAAAAATAGTCATTTTCAACATATTTTTTATACATGTCCTCGGTATATCTTGTTATGTTCTTACCATACTCTGGATAATCAAACCCCAGTAGTTCTGCTACCTCTTTGGACACTTCCCTGAACAATTGGTGGCATATAAATAATGACTTCCAAATATTTTCATAGGAATCCATGCGATATGTAGATAATAATCTATTCCATAGATCTTCATCAATGTATTTGTTAATATACTTATAATTTTTCCCAACACTTAATGAAAATTCTGTCTTTATCCCAACCTTCCACGACATCATCCTAAGTAGTTCAAACCGTAGAATCTGGTTCAGATGATCGATTGCAAACAGTATCTCTTTGCGGCACAATCCTTTAATAACATAAGGTGTTACATTCCAAAATTCATTGCAGCAATCATCATACTCCCTTGCGCTTGGCTTTCTTACATGATAATCTATATCAGTCGGAACTATGTCCCTTTTATTCTACATCTTTATCATTAGAACCTTTATAGTTATCGCCCTTAGTATATCTACTCTCAGGCATAGTAGATCATTTATGTATCATCAATAGCATAGTAGAAATCCTTTCATCAGTGAATAAAATATCTCTTATA | [Enterococcus faecalis strain E505 lsa(E)-carrying multidrug resistance gene cluster, partial sequence](https://blast.ncbi.nlm.nih.gov/Blast.cgi#alnHdr_1039262215) | 97 | 97 | [KX156279.1](https://www.ncbi.nlm.nih.gov/nucleotide/KX156279.1?report=genbank&log$=nucltop&blast_rank=15&RID=B9S9XZP2015) |
| [Streptococcus agalactiae strain SGB76 insertion sequence IS1216E transposase (tnp) gene, complete cds; and aminoglycoside 6-adenylyltransferase (aadE), putative adenine phosphoribosyltransferase (apt), putative spectinomycin adenylyltransferase (spc), hypothetical proteins, ABC transporter, lincosamide nucleotidyltransferase (lnuB), transposase-like ISL3 family protein (transposase), hypothetical protein, and putative methyltransferase protein genes, complete cds](https://blast.ncbi.nlm.nih.gov/Blast.cgi#alnHdr_576639548) | 97 | 97 | [KF772204.1](https://www.ncbi.nlm.nih.gov/nucleotide/KF772204.1?report=genbank&log$=nucltop&blast_rank=29&RID=B9S9XZP2015) |
| [Enterococcus faecium strain P23 plasmid pXD4 ErmB (ermB), AadE (aadE), hypothetical protein, Spw (spw), LsaE (lsaE), LnuB (lnuB), and Tnp (tnp) genes, complete cds](https://blast.ncbi.nlm.nih.gov/Blast.cgi#alnHdr_565363047) | 97 | 97 | [KF421157.1](https://www.ncbi.nlm.nih.gov/nucleotide/KF421157.1?report=genbank&log$=nucltop&blast_rank=30&RID=B9S9XZP2015) |
| ***cat*** | *L. ingluviei* 22eI | GRKWWCTGTAGATAGCGGTAATATATTGAATTACCTTTATTAATGAATTTTCCTGCTGTAATAATGGGTAGAAGGTAATTACTATTATTATTGATATTTAAGTTAAACCCAGTAAATGAAGTCCATGGAATAATAGAAAGAGAAAAAGCATTTTCAGGTATAGGTGTTTTGGGAAACAATTTCCCCGAACCATTATATTTCTCTACATCAGAAAGGTATAAATCATAAAACTCTTTGAAGTCATTCTTTACAGGAGTCCAAATACCAGAGAATGTTTTAGATACACCATCAAAAATTGTATAAAGTGGCTCTAACTTATC  CCAATAACCTWAA | [Staphylococcus sciuri pC194-like catA gene for type A-9 chloramphenicol O-acetyltransferase, complete CDS](https://blast.ncbi.nlm.nih.gov/Blast.cgi#alnHdr_1035501782) | 96 | 99 | [NG_047580.1](https://www.ncbi.nlm.nih.gov/nucleotide/NG_047580.1?report=genbank&log$=nucltop&blast_rank=52&RID=B87J9AFV015) |
|  |  | [Staphylococcus aureus rep, cat genes for replication protein, chloramphenicol acetyltransferase, complete cds, strain: OC8](https://blast.ncbi.nlm.nih.gov/Blast.cgi#alnHdr_757805036) | 96 | 99 | [AB982227.1](https://www.ncbi.nlm.nih.gov/nucleotide/AB982227.1?report=genbank&log$=nucltop&blast_rank=69&RID=B87J9AFV015) |
| ***int-Tn*** | *L. salivarius* 30aI | GAGAGTTAMGAAAGAMTTCATGATGGTATTGATGTTGTAGGAAAGAAAATGACACTCTGCCAGCTTTACGCAAAACAGAACGCTCAAAGACCAAAGGTTAGAAAAAACACTGAAACTGGACGCAAATATCTTATGGATATTTTGAAGAAAGACAAGTTAGGTGTAAGAAGTATTGACAGTATTAAGCCMTCAGACGCTAAAGAATGGGCTATTAGAATGAGTGAAAATGGTTATGCTTATCAAACCATCAATAACTACMAACGTTCTTTAAAGGCTTCMTTCTATATTGCTATACAAGATGATTGTGTTCGGAAGAATCCATTTGACTTTCAACTGAAAGCAGTTCTTGATGATGATACTGTCCCTAAGACCGTACTAACAGAAGAACAGGAAGAAAAACTGTTAGCCTTTGCAAAAGCTGATAAAACCTACAGCAAAAATTATGATGAAATTCTGATACTCTTAAAAACAGGTCTTCGTATTTCAGAGTTTGGTGGTTTGACACTTCCAGATTTAGATTTTGAGAATCGTCTTGTCAATATAGACCATCAGCTATTGAGAGATACTGAAATTGGGTACTACATTGAAACACCAAAGACCAAAAGTGGCGAACGTCAAGTTCCTATGGTTGAAGAAGCCTATCAAGCATTTAAGCGAGTGTTAGCGAATCGAAAGAATGATAAGCGTGTTGAGATTGATGGATATAGTGATTTCCTCTTTCTTAATAGAAAGAACTATCCAAAAGTGGCAAGTGATTACAACGGCATGATGAAAGATCTTGTTAAGAAATACAATAAGTATAACGAGGATAAATTGCCACACATCACTCCACATAGTTTGCGACATACATTCTGTACCAACTATGCAAATGCAGGAATGAATCCAAAGGCATTACAGTACATTATGGGACATGCTAATATAGCCATGACGCTGAACTATTACGCACATGCAACATTCGATTCTGCAATGGCAGAAATGAACGCTGATAAGGASAAGCCCAGCGGGGRGYSWMWMATTCATGC | [Streptococcus suis integrative and conjugative element ICESsu05SC260 mobile element, complete sequence](https://blast.ncbi.nlm.nih.gov/Blast.cgi#alnHdr_1036162460) | 97 | 99 | [KX077888.1](https://www.ncbi.nlm.nih.gov/nucleotide/KX077888.1?report=genbank&log$=nucltop&blast_rank=19&RID=B83V0RYF014) |
| [Streptococcus pneumoniae Tn916-type integrative and conjugative element, strain 11930](https://blast.ncbi.nlm.nih.gov/Blast.cgi#alnHdr_321157404) | 97 | 99 | FR671416.1 |
| [Bacillus subtilis transposon Tn916, complete sequence](https://blast.ncbi.nlm.nih.gov/Blast.cgi#alnHdr_760459614) | 97 | 99 | [KM516885.1](https://www.ncbi.nlm.nih.gov/nucleotide/KM516885.1?report=genbank&log$=nucltop&blast_rank=49&RID=B83V0RYF014) |
| ***int-Tn*** | *E. faecalis* 3 | GSGAGGTWMGAAAGAMTTCATGATGGTATTGATGTTGTAGGAAAGAAAATGACACTCTGCCAGCTTTACGCAAAACAGAACGCTCAAAGACCAAAGGTTAGAAAAAACACTGAAACTGGACGCAAATATCTTATGGATATTTTGAAGAAAGACAAGTAGGTGTAAGAAGTATTGACAGTATTAAGCCMTCAGACGCTAAAGAATGGGCTATTAGAATGAGTGAAAATGGTTATGCTTATCAAACCATCAATAACTACMAACGTTCTTTAAAGGCTTCATTCTATATTGCTATACAAGATGATTGTGTTCGGAAGAATCCATTTGACTTTCAACTGAAAGCAGTTCTTGATGATGATACTGTCCCTAAGACCGTACTAACAGAAGAACAGGAAGAAAAACTGTTAGCCTTTGCAAAAGCTGATAAAACCTACAGCAAAAATTATGATGAAATTCTGATACTCTTAAAAACAGGTCTTCSTATTTCAGAGTTTGGTGGTTTGACACTTCCAGATTTAGATTTTGAGAATCGTCTTGTCAATATAGACCATCAGCTATTGAGAGATACTGAAATTGGGTACTACATTGAAACACCAAAGACCAAAAGTGGCGAACGTCAAGTTCCTATGGTTGAAGAAGCCTATCAAGCATTTAAGCGAGTGTTAGCGAATCGAAAGAATGATAAGCGTGTTGAGATTGATGGATATAGTGATTTCCTCTTTCTTAATAGAAAGAACTATCCAAAAGTGGCAAGTGATTACAACGGCATGATGAAAGGTCTTGTTAAGAAATACAATAAGTATAACGAGGATAAATTGCCACACATCACTCCACATAGTTTGCGACATACATTCTGTACCAACTATGCAAATGCAGGAATGAATCCAAAGGCATTACAGTACATTATGGGACATGCTAATATAGCCATGACGCTGAACTATTACGCACATGCACATTCGATTCTGCATGCAGAAATGAACGCTGAATAAGASAAAAAGMRGCGGGSWKCKAYMAGTCGGGTCTATTATGASAKKCTCCAATRATGATTGGAATGCTTAGAATCATGCGTACATTGCATAGTGACAAGGAAKATSKATCTAACTAG | [Streptococcus suis integrative and conjugative element ICESsu05SC260 mobile element, complete sequence](https://blast.ncbi.nlm.nih.gov/Blast.cgi#alnHdr_1036162460) | 88 | 99 | [KX077888.1](https://www.ncbi.nlm.nih.gov/nucleotide/KX077888.1?report=genbank&log$=nucltop&blast_rank=19&RID=B87U3ZD0014) |
| [Bacillus subtilis transposon Tn916, complete sequence](https://blast.ncbi.nlm.nih.gov/Blast.cgi#alnHdr_760459614) | 88 | 99 | [KM516885.1](https://www.ncbi.nlm.nih.gov/nucleotide/KM516885.1?report=genbank&log$=nucltop&blast_rank=49&RID=B87U3ZD0014) |
