# Supplementary material for: Identification and antibiotic susceptibility of lactobacilli isolated from turkeys
Source: BMC Microbiol. 2018 Oct 29;18:168. doi: 10.1186/s12866-018-1269-6 (PMC6206647; doi:10.1186/s12866-018-1269-6)
Supplement: Supplementary file 3 — Table S3. Containing sizes (bp) of restriction fragments obtained by cleavage of 16S rDNA amplicons of reference and wild-type isolates of Lactobacillus. (DOC 31 kb) [file 12866_2018_1269_MOESM3_ESM.doc]

Additional file 3: Table S3. Sizes (bp) of restriction fragments obtained by cleavage of 16S rDNA amplicons of reference and wild-type isolates of *Lactobacillus*. The values in brackets refer to single wild isolates.

| Phylogenetic group | Species | Size (bp) of restriction fragments found in wild strains | | |
| --- | --- | --- | --- | --- |
|  |  | ***Mse*I** | ***Mbo*I** | ***Alu*I** |
| *L. delbrueckii* | *L. johnsonii* | **126, 140, 253, 910** | 180, 324, 920 | 180, 205, 250, 410 |
|  | *L. gasseri* | **126, 220, 405, 574, 670** | 180, 324, 920 | 180, 205, 250, 410 |
|  | *L. crispatus* | 125, 210, 250, 410 | **117, 172, 190, 930** | 182, 210, 430 |
|  | *L. ultunensis* | 125, 210, 250, 410 | **117, 172, 930** | 182, 210, 430 |
| *L. reuteri* | *L. oris* | 120, 180, 240, (420), 626 | 165, 180, 325, (480), 920 | **100, 187, 280, 630** |
|  | *L. antri* | 120, 180, 240, 626 | 165, 180, 325, 920 | **82, 100, 180, 200, 270, 415** |
